# The development and implementation of a regional network of physiotherapists for exercise therapy in patients with peripheral arterial disease, a preliminary report

**DOI:** 10.1186/1472-6963-5-49

**Published:** 2005-07-12

**Authors:** EM Willigendael, BLW Bendermacher, C van der Berg, RJThJ Welten, MH Prins, RA Bie de, JAW Teijink

**Affiliations:** 1 Atrium Medical Centre, Department of Surgery, Division of Vascular Surgery, Heerlen, The Netherlands; 2 Atrium Medical Centre, Department of Cardiology, Heerlen, The Netherlands; 3 Department of Epidemiology, University of Maastricht / KEMTA, Maastricht, The Netherlands

## Abstract

**Background:**

Exercise therapy (ET) is the main conservative and proven effective treatment of patients with intermittent claudication. Currently, the most frequent exercise prescription is a single 'go home and walk' advise, without supervision or follow-up. There is no evidence to support the efficacy of this advise and compliance is known to be low. Therefore, a systematic approach was used to guarantee quality and standardisation of treatment, optimal guideline adherence and improved of inter-professional communication between vascular surgeons and physiotherapists. In this preliminary report we would like to outline the steps taken for the development and implementation of the Network Exercise Therapy Parkstad

**Methods:**

In October 2003 all 59 regional physiotherapy practices were invited to attend a symposium regarding ET in a physiotherapeutic setting. Attending physiotherapists interested in providing ET and willing to follow a certified course on ET, were asked to register. Three tastkgroups were formed to accomplish the set targets: Exercise therapy education, Exercise therapy implementation and continuity, and Inter-professional communication in the Parkstad region.

**Results:**

In total 27 physiotherapists, from 22 different practices followed the educational program and are now trained and accredited to provide ET according to the guideline of the Royal Dutch Society for Physiotherapy. A web-based database wasdesigned to contain information on disease specific items provided by the vascular surgery department, and aspects with respect to ET registered by the physiotherapist. The information is regularly updated and available online. Access tothe database is restricted to vascular surgeons and physiotherapists in the network. The secondary purpose of the database is to register essential benchmark data for future analysis of ET in a physiotherapeutic setting in the Netherlands and to enable physiotherapists continuous feedback on patient performance. A triage system was developed to detect patients with a compromised cardiac history. This group receives ET at the in-hospital department of revalidation with the possibility of immediate consultation of a cardiologist in case of cardiac complications or even CPR.

**Conclusion:**

The Network Exercise Therapy Parkstad of supervised ET is the first initiative in the Netherlands to provide ET close to the patient's home environment. With the implementation of supervised ET in an outpatient physiotherapeutic setting for all eligible patients with symptomatic PAD, the access to care has been improved. A web-based communication system provides physiotherapists and vascular surgeons with all the necessary and continues updated patient information. Future research, currently in progress, will investigate the therapeutic benefits and cost-effectiveness of exercise therapy in a physiotherapeutic setting.

## Background

Patients with peripheral arterial disease (PAD), with intermittent claudicationas the predominant clinical symptom, experience muscle aching or cramp during walking, secondary to muscle ischemia in the calf, thigh or buttocks. PAD is a manifestation of systemic atherosclerosis. The treatment of patients with PAD stage II (according to Fontaine) consists of vascular risk factor management, smoking cessation and exercise therapy (ET).

ET is the main conservative treatment for patients with intermittent claudication and proven effective.[1] The psychological, metabolical, and mechanical alterations that occur during the periods of exercise stimulate an adaptive response that ultimately reduces the symptoms of intermittent claudication. Besides the improvement in maximal walking distance, ET contributes to an increase in quality of life and a decrease in the number of vascular interventions.[2] Furthermore, with adequate ET, hypertension, hypercholesterolaemia, overweight, and diabetes, if present, are better regulated. These positive results of exercise therapy have been observed during training programs in hospitals or rehabilitation clinics. [3,4] However, major disadvantages of these hospital-based programs are that they are costly, their availability is limited, and patients are taken out of their family and working environment.

Currently, the most frequent exercise prescription for patients with PAD in theNetherlands, and considered best practice according to the Dutch general practitioners and vascular surgeons claudication intermittens guidelines, is a single'go home and walk' advise, without supervision or follow-up. A leaflet on unsupervised ET provided by the Vascular Patients Society is available. There is no evidence to support the efficacy of this advise and compliance is known to be low. [5,6] In studies comparing the 'go home and walk' advise to supervised ET, a clear advantage for supervised ET was present. [6-9] The ineffectiveness of home based ET is to a large extent caused by the pain caused by ET and the subsequent reluctance to exercise again. Factors like fear of pain, inadequate knowledge and poor general condition, contribute to the difficulty to start, sustain and maintain ET. ET in a physiotherapeutic setting has the benefits of adequate coaching and provides the stimulation and supervision deemed necessary to provide an advantage over the 'go home and walk' advice. Furthermore, the close contact with patients during ET in a physiotherapeutic setting provides coaching in the necessary changes in life-style, like weight control and smoking cessation.

The first national physiotherapeutic guideline on ET was issued in December 2003 by the Royal Dutch Society for Physiotherapy.[10] This guideline enablesthe facilitation of professional ET in the future. However, a national cross-sectional survey performed among 265, randomly selected Dutch physiotherapists showed deficits in the physiotherapists theoretical and practical skills regarding ET.[11] This illustrates again the fact that the existence of a guideline does not warrant optimal care or automatic goal attainment.

Therefore, a systematic approach was used to guarantee quality and standardisation of treatment, optimal guideline adherence and improved inter-professional communication between vascular surgeons and physiotherapists.

To improve the current care for patients with intermittent claudication, we setthe following goals:

• Optimalisation of the current knowledge and skills of the physiotherapists

• Improve the screening of patients allegeable for ET and to provide a safety-net for patients with a high cardiovascular risk

• ET should be accessible close to the patients home address

• Implementation of a continues monitoring and exchange of inter-professional patient information system

To achieve these goals, the so-called Network Exercise Therapy Parkstad steering committee was founded in October 2003. This steering committee consisted of avascular surgeon, three nurse practitioners of the vascular surgery department, an epidemiologist, five physiotherapists and a research fellow of the vascular surgery department. To our knowledge this concept is innovative for the Netherlands and abroad. Before future research will commence, we would like to outlinethe steps taken for the development and implementation of the Network ExerciseTherapy Parkstad

## Methods

### Exercise therapy network development

In October 2003 all 59 regional physiotherapy practices were invited to attend a symposium regarding ET in a physiotherapeutic setting. The symposium covered topics on the theoretical background, therapeutic benefits, and an introductionto the new Royal Dutch Society for Physiotherapy guideline on ET. An important issue of the symposium was an open discussion on the formation of the Network Exercise Therapy Parkstad to facilitate supervised ET for patients referred by vascular surgeons from the Atrium Medical Centre. At the end of the symposium, attending physiotherapists interested in providing ET, and in the possession of, or the willingness to acquire, a treadmill, and willing to follow a certified course on ET, were asked to register. From this group of physiotherapists we planned to invite five physiotherapists to join the aforementioned steering committee. Our aim was to obtain both physiotherapists from the Department of Revalidation in our hospital as well as regional physiotherapists.

Three taskgroups within the steering committee were formed to accomplish the set targets:

• Exercise therapy education

• Exercise therapy implementation and continuity

• Inter-professional communication in the Parkstad region

### Exercise therapy education

To overcome the noted knowledge deficiencies of ET, this taskgroup organised anaccredited two-day 'in-hospital' educational program on ET provided by the Royal Dutch Society for Physiotherapy and the Dutch Paramedic Institute. This course had already been developed, and provides the necessary knowledge and skills to give professional supervised ET. The course covered the necessary theoreticalbackground, and practical skills to provide ET. Three months later, this coursewas followed by a one day follow-up training. This training took place to exchange practical experiences, and to pay extra attention to the use of clinimetrics, standardisation and optimisation of the protocol, as well as getting an introductory session on the use of the electronic patient file in the web-based database.

### Exercise therapy implementation and continuity

**Figure 1:**
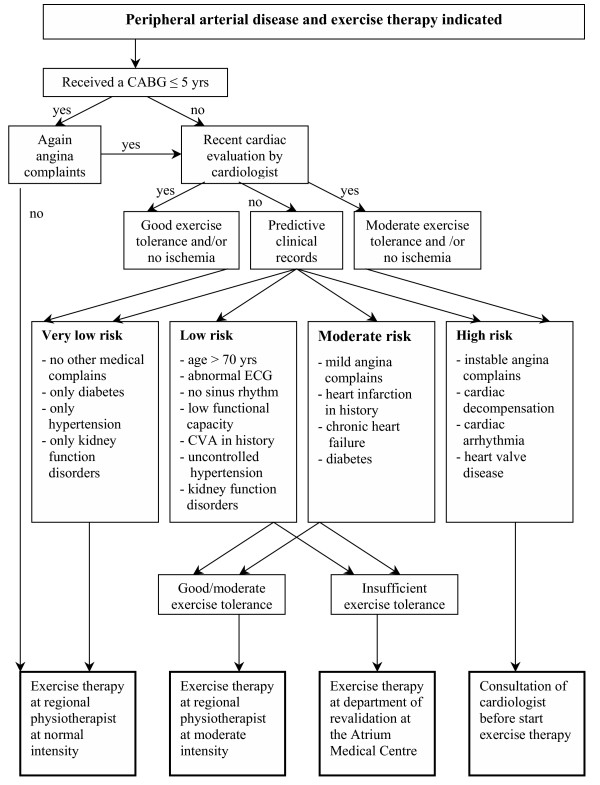
Decision tree to triage patients with a compromised cardiac history.

In some patients with PAD, ET in an outpatient physiotherapeutic setting interferes with the present high cardiovascular risk. To provide ET for patients witha compromised cardiac history in a relatively safe environment, the second taskgroup developed a triage system to filter these patients prior to the ET prescription. In close collaboration with the department of cardiology, a decision tree has been developed.(Figure 1) This enabled the nurse practitioners at the department of vascular surgery to decide if ET can be performed in an outpatient setting or at the in-hospital department of revalidation with the possibility of immediate consultation of a cardiologist in case of cardiac complications or even CPR.

The aforementioned leaflet on unsupervised ET of the Vascular Patients Society, which has been used throughout the Netherlands for over 10 years, needed revision. The Vascular Patients Society gave us the opportunity to suggest some alterations in this leaflet to make it compliant with the possibility of supervised ET. In this revised version attention was given to the new insights on PAD, risk factor management and supervised as well as unsupervised ET.

ET requires a life-long adaptation of the patient's life-style, in which a continuation of daily walking plays an essential role. ET in a physiotherapeutic setting is only covered by medical insurance in the Netherlands for the durationof one year. To support the patient with the necessary life-style changes, the 'Regional Exercise Therapy Walking group Parkstad' has been formed for patientsto enrol after the termination of the supervised ET after a year.

### Inter-professional communication in the Parkstad region

The last taskgroup tried to solve the so far absent or at least insufficient communication between the department of vascular surgery and physiotherapy practices. In close collaboration with the University of Maastricht, department of epidemiology, a web-based database was developed. The purpose of this database was twofold. The primary purpose was to facilitate adequate information exchange between vascular surgeons/nurse-practitioners and physiotherapists. The database was designed to contain information on disease specific items like the extentof PAD, present risk factor and co-morbidity provided by the vascular surgery department. The physiotherapist registered aspects with respect to the ET, like therapy progress and difficulties as well as patient compliance. The information is regularly updated and online available. The secondary purpose of the database is to register essential data for future analysis of ET in a physiotherapeutic setting and to enable physiotherapists to receive continuous feedback (benchmark data) on their performance. Furthermore, this database has been designed to be implemented nationwide, and when accomplished, a substantial and important level of evidence on the value of physiotherapy will be available. Similar databases are currently being developed for other therapies in a physiotherapeutic setting.

## Results

### Exercise therapy network implementation

#### Exercise therapy education

64 physiotherapists attended the symposium on the future of ET in the Parkstad region from 45 regional physiotherapy practices. Five physiotherapists, two from the Department of Rehabilitation, Atrium medical centre, and three regional physiotherapists joined the steering committee. In total 27 physiotherapists, from 22 different practices followed the educational program and are now trained and accredited to provide ET according to the guideline.

### Exercise therapy implementation and continuity

**Figure 2:**
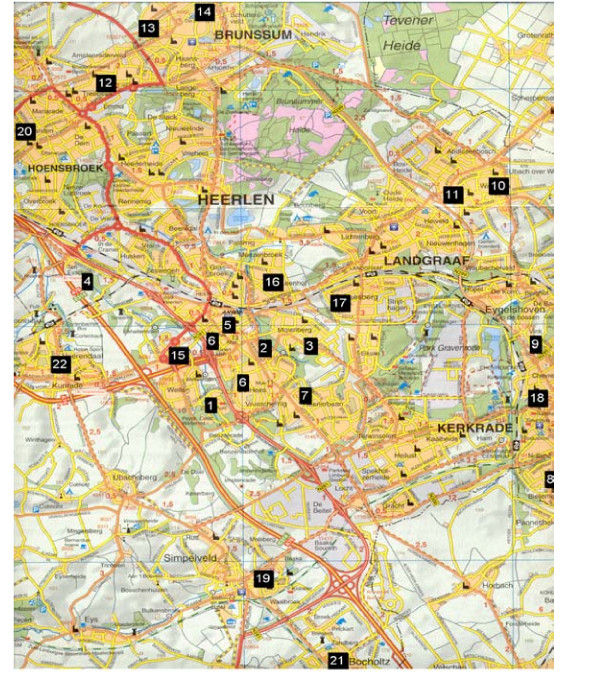
Regional physiotherapy practice participation and distribution.

Patients with a serious cardiac medical history receive ET in a hospital setting, but the vast majority is referred to a local physiotherapist. (Figure 2) Therevised ET leaflet of the Vascular Patients Society has been nationally released in the autumn of 2004. Students of the regional physiotherapy school, under supervision of a steering committee physiotherapist are coaching the 'Regional Exercise Therapy Walking group Parkstad'. The primary goal of this walking group is to stimulate patientsto remain physically active after the termination of the supervised ET. The walking group organises regular walks every 14 days, in which life style changes, like smoking cessation, weight control, and contacts with other patients play a central role.

### Inter-professional communication in the Parkstad region

The web based database provides vascular surgeons and physiotherapists with essential information. Before the patients are scheduled for a repetitive appointment at the hospital the nurse practitioner has access to all therapy related information. The same principle applies to the physiotherapist who receives all the medical updates. With this information the physiotherapist is better equipped to make an adequate judgement on the exercise capacity of the patient and thevascular surgeon can, based on therapy progress and compliance, make an adequate judgement on the necessity of a vascular intervention. Access to the databaseis restricted to vascular surgeons and physiotherapists in the network.

In June 2004 the Network Exercise Therapy Parkstad has started. It is the firstnational network to provide supervised ET in a physiotherapeutic setting close to the patients home. All patients with symptomatic PAD (Fontaine stage II) presented at the vascular surgery outpatient clinic who are considered for maximalnon-invasive treatment, are now primarily referred to one of the physiotherapists in the Network Exercise Therapy Parkstad for supervised ET.

## Discussion

In the 1998 report of the Dutch Heart Foundation on obstacles in vascular care, the inadequate ET facilities and infrastructure was noted.[12] Recommendations were made to stimulate the use of ET and to develop an ET infrastructure. Six years later the Network Exercise Therapy Parkstad of supervised ET is the first initiative to provide ET close to the patient's home. Supervised ET has been provided on a small scale in the Netherlands, primarily hospital based. Withthe implementation of supervised ET in an outpatient physiotherapeutic setting for patients with symptomatic PAD, an attempt has been made to improve the quality of care, and the communication between the different care providers.

Less than half (49%) of the physiotherapists that attended the symposium, eventually jointed the network. This seems disappointing, but taken into account that only 30% of the Dutch practices is in the possession of the required treadmill, the results were better than expected. Furthermore, the majority of practices are already specialised in for instance, cardiac rehabilitation, pulmonary diseases, sport-physiotherapy or neurological disorders, leaving little room for an additional (and time consuming) speciality. The 22 practices that were capable to meet the set requirements, cover the region well and are highly motivated.

### Network Exercise Therapy Parkstad

ET in a physiotherapeutic setting is more expensive than the 'go home and walk' advise. However, besides the increase in maximal walking distance, the prognosis of general health (diabetes, high cholesterol, hypertension, bodyweight, blood pressure, quality of life, life-style changes like smoking, and an inactive life-style) may improve. Potential differences in costs are likely to result from less morbidity and medical consumption related to PAD complications, vascular surgery, and associated cardiovascular diseases. It is expected that these savings will exceed the costs of ET in a physiotherapeutic setting. However, a true cost-effectiveness analysis should answer this question. On the other hand, ET in hospitals or rehabilitation clinics, as have been practiced abroad, is more costly. The availability of ET close to the patient's home address is one ofthe advantages of the Network Exercise Therapy Parkstad and is most likely to substantially improve therapy participation and compliance. The group that has not the benefit of supervised ET 'around the corner', is the 'safety-net group' for patients with a high cardiovascular risk. In the previous situation, these patients would not be engaged in ET or suffer an increased risk during unsupervised ET. Providing ET in a hospitalised setting enables patients to slowly reclaim their health in a protective environment. The developed decision tree (which has been incorporated in the web-based database), enables also vascular nursepractitioners to identify these high-risk patients.

### Inter-professional communication in the Parkstad region

The Network Exercise Therapy Parkstad requires a different way of working and an intensified manner of communication between the department of vascular surgery and the participating physiotherapists. Working with the web-based database is, compared to the old situation, a new way of working and relatively time consuming. Future research will show the impact and consequences of this database.

## Conclusion

This preliminary report describes the development and implementation of the Network Exercise Therapy Parkstad. The main goal of this regional network was to improve the access to care, and the communication between the different care providers. Future research, currently in progress, will investigate the therapeutic benefits and the cost-effectiveness of exercise therapy in a physiotherapeutic setting. The Network Exercise Therapy Parkstad is now operational and general practitioners have been invited to join and refer patients from the primary care setting. To guide other interested regions in similar projects an extensive implementation guideline is available.

## Competing interests

The author(s) declare that they have no competing interests.

## Authors' contributions

EW concept development and implementation of network and writing of the manuscript. BB management and implementation of network, preparation of manuscript. CB development and implementation of cardiac decision tree. RW concept implementation and critical review of manuscript. MP participated in the design of the concept and draft of manuscript. RB development of database, concept implementation and critical review of manuscript. JT primary concept development and implementation of network and writing of the manuscript. All authors read and approved the final manuscript.

## Pre-publication history

The pre-publication history for this paper can be accessed here:


